# Supplementary material for: Splice-Junction-Based Mapping of Alternative Isoforms in the Human Proteome
Source: Cell Rep. Author manuscript; Available in PMC 2020 Jan 15. (PMC6961840; doi:10.1016/j.celrep.2019.11.026)
Supplement: 2 [file NIHMS1546469-supplement-2.zip › DF1/PXD006675/CardiacFibroblast/CardiacFibroblast_14_NECAB3_EGLQSGPVCDALPAAG.pdf]

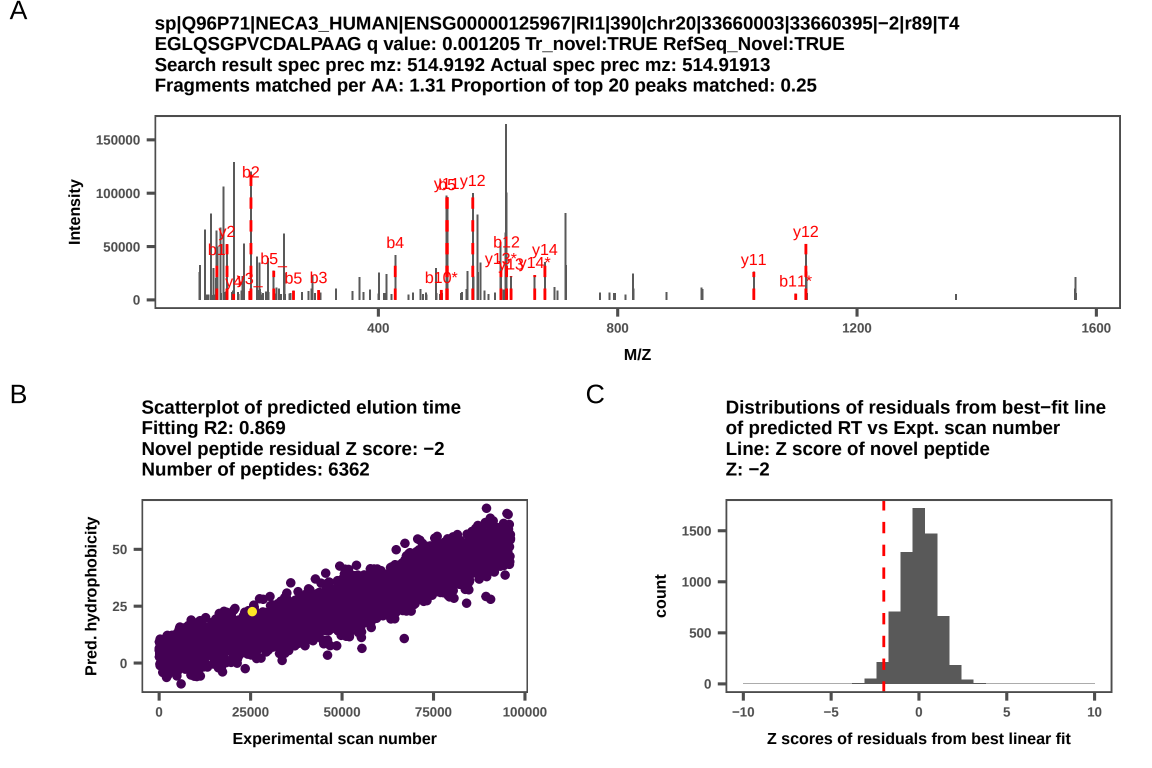
A
sp|Q96P71|NECA3_HUMAN|ENSG00000125967|RI1|390|chr20|33660003|33660395|−2|r89|T4
EGLQSGPVCDALPAAG q value: 0.001205 Tr_novel:TRUE RefSeq_Novel:TRUE
Search result spec prec mz: 514.9192 Actual spec prec mz: 514.91913
Fragments matched per AA: 1.31 Proportion of top 20 peaks matched: 0.25
Intensity
150000
100000
50000
0
b2
y2
b1
b5_
y4
y3_
b5
b3
b4
b10*
y12
b12
y13*
y13
y14*
y14
y11
y12
b11*
400
800
1200
1600
M/Z
B
Scatterplot of predicted elution time
Fitting R2: 0.869
Novel peptide residual Z score: −2
Number of peptides: 6362
Pred. hydrophobicity
50
25
0
0
25000
50000
75000
100000
Experimental scan number
C
Distributions of residuals from best−fit line
of predicted RT vs Expt. scan number
Line: Z score of novel peptide
Z: −2
count
1500
1000
500
0
−10
−5
0
5
10
Z scores of residuals from best linear fit
